# Impact of plateau pikas (*Ochotona curzoniae*) on soil properties and nitrous oxide fluxes on the Qinghai-Tibetan Plateau

**DOI:** 10.1371/journal.pone.0203691

**Published:** 2018-09-27

**Authors:** Yan Zhou, Shengwu Jiao, Nana Li, John Grace, Meng Yang, Cai Lu, Xuemeng Geng, Xinwei Zhu, Li Zhang, Guangchun Lei

**Affiliations:** 1 Southern Modern Forestry Collaborative Innovation Center/College of Biology and the Environment, Nanjing Forestry University, Nanjing, China; 2 School of Nature Conservation, Beijing Forestry University, Beijing, China; 3 Research Institute of Subtropical Forestry, Chinese Academy of Forestry, Fuyang, China; 4 School of Geosciences, University of Edinburgh, Edinburgh, United Kingdom; 5 Aba Prefecture Research Institute of Forestry Science and Technology, Aba, China; Sichuan University, CHINA

## Abstract

This paper demonstrates the impact of an endemic fossorial animal, plateau pika (*Ochotona curzoniae*), on soil properties and N_2_O flux at the Zoige Wetland. Pika burrow and control sites without disturbance by pika were selected to measure the soil water content, bulk density, soil organic matter (SOM), NH_4_-N content and NO_3_-N content in August 2012. N_2_O fluxes were measured with static opaque chambers at these sites in June and August 2012. Pika burrowing altered soil aeration by transferring deeper soil to the surface and by constructing underground burrows, which significantly increased bulk density, and reduced soil water content, SOM and NH_4_-N content at 0–10 cm and 10–20 cm soil depth. N_2_O flux had a significant correlation with bulk density, SOM and NH_4_-N content. Pika burrowing significantly influenced N_2_O flux by increasing N_2_O flux at the control site from near zero to 0.063±0.011 mg m^-2^ h^-1^. Our findings described how pika burrowing influences the soil traits and significantly increases the principal greenhouse gas N_2_O emission. As plateau pika was commonly considered as a pest, our findings give a novel clue to effectively manage populations of plateau pika on the Qinghai-Tibet Plateau from the perspective of greenhouse gas emission.

## Introduction

Animal activity plays an important role in modulating biogeochemical cycles of water, carbon and nutrients, primarily through disturbance and altering soil properties [1,2]. In meadow ecosystems, burrowing activities of rodents, lagomorphs and other herbivorous mammals cut grass roots, dig and transfer soils from the subterranean to the surface, and excavate mounds[3,4]. These activities can profoundly change the structure and functional dynamics of meadow soils [5–7]. The altered soil properties and changed underground aerobic/anaerobic environment are expected to influence carbon and nitrogen cycles and the ecosystem release of greenhouse gases to the atmosphere [8–10].

Nitrous oxide (N_2_O) is an important greenhouse gas whose warming potential is 298 times that of the same mass of carbon dioxide (CO_2_) [11]. The concentration of N_2_O in the atmosphere has increased from a preindustrial concentration of approximately 270±7 ppb in 1750 to 324.2 ppb in 2011 [12], and anthropogenic and natural soil disturbance has been an important sources for the increased atmospheric N_2_O [13,14]. N_2_O is produced in soils via the chemical processes of denitrification and nitrification, which are regulated by soil moisture, soil nitrogen (N) content, and temperature [15]. Burrowing activities can definitely influence N_2_O emissions through changing soil physical and chemical properties and modifying soil aeration conditions [16,17]. However, direct evidence on how and to what extent lagomorph burrowing activities impact soil properties and N_2_O emission of meadow ecosystems is still obscure [6]. Although the plateau pika is considered to be a harmful mammal for its burrowing and foraging, previous work has revealed that pikas are important for maintaining or restoring native plant communities by improving the soil quality [18]. Furthermore, due to the alteration of soil properties, monitoring the greenhouse gas emission in the increasing distribution area of pika burrow will improve understanding ecological role of pikas and then enhance the wildlife management in the Zoige Wetland.

In this study, we quantitatively investigate the impacts of the burrowing activities of plateau pika (*Ochotona curzoniae*) on soil properties and N_2_O emissions of an alpine meadow ecosystem in southeastern Qinghai-Tibetan Plateau. Plateau pika is an endemic species to the Qinghai-Tibetan Plateau widely distributed from the Himalayan to the Hengduan Mountains [19]. These animals live underground throughout their lifetime and are active burrowers, sometimes emerging for mating and food collection on the ground [20]. The burrowing reduces grass yield essential for local husbandry, e.g. yaks, goats etc., for which plateau pikas have been viewed as pests and are subject to pest controls [21]. However, others argue that plateau pikas are keystone species performing essential ecosystem roles, such as increasing plant diversity, in the alpine meadow ecosystem [22]. A better understanding of the role of plateau pikas’ burrowing activity on soil properties and N_2_O, therefore, is important for assessing the ecosystem role of this endemic species.

## Material and methods

This study was authorized by the Management Bureau of Zoige National Nature Reserve. The field study did not involve endangered or protected species, and no specific permissions were required for the study.

### Site description and site selection

This study was conducted at Lake Huahu (33.5°N, 102.5°E, 3430 m a.s.l.), the Sichuan Zoige wetland National Nature Reserve, approximately 40 km north of Ruoergai County town, Sichuan province, China. It has a typical Tibetan plateau cold climate. The annual mean precipitation is 650 mm and the mean temperature is 1.7 °C with a three-month growing season from June to August where most of the precipitation occurs [23].

As the groundwater table descends with the increase of the distance to the Huahu Lake water surface, plateau pikas got more possibility to inhabit in the area with low groundwater table to reduce the risks of being inundated. According to the groundwater table and distribution of pika burrow, two plots were selected for N_2_O emission studies. The distance from plot PIK (33.929°N, 102.820°E; 3439m a.s.l.) to plot LIT (33.918°N, 102.818°E; 3435m a.s.l.) is approximately 1200m. The plot LIT is very close to the Huahu Lake with a higher groundwater table than that at the plot PIK. Three sets of samples were taken the BUR set at the plot PIK was placed on pika burrow with the whole burrow involving in the chamber base, to measure N_2_O emission from pika burrow., whilst the CON set at the plot PIK and the LIT set at the plot LIT representing the control samples for BUR measure N_2_O emission from meadow without burrow on the surface. When measuring, samples of BUR, CON and LIT were selected along sampling lines which are determined by random start points and random directions. Each sample located from others at least 5 m. *Carex muliensis* and *Potentilla anserina* are the predominant plant species covering about 50%-90% of the area at the sites LIT, CON and BUR. Aboveground vegetation was removed by cutting down along the ground surface to measure N_2_O flux without the impact of aboveground vegetation.

### N_2_O flux measurement

Due to the much lower ambient temperature, Greenhouse gas fluxes emitted during the non-growing season stay at low magnitude on the Qinghai-Tibet Plateau [9], while pika burrowing is more active in growing season. Consequently, the impact of pika burrowing on N_2_O flux mainly occurs in the short growing season. To precisely evaluate impact of pika burrowing, N_2_O flux was measured in June and August 2012. The static opaque chamber technique was used to determine the N_2_O flux [24]. When measuring N_2_O flux, six chambers (volume: 125 L, surface area: 0.25 m^2^) were placed randomly at the site LIT and CON. When measuring N_2_O flux at the site BUR, five chambers were placed on pika burrows that cover about 30–50% surface area of each chamber.

The stainless steel-chambers were surrounded by polyethylene foam to avoid any temperature fluctuations inside. Two fans were installed in the chamber to mix the air. Each chamber has a 20 cm length base which was installed 15 cm into the ground to mediate pressure pumping in chamber during wind gusts. Furthermore, each chamber was installed at least 2 days before gas samples collecting each time to avoid disturbing to greenhouse gas production in soil. Using 100 ml polypropylene syringes, four gas samples (200ml of each) from each chamber were taken at 10 minutes intervals over a 30 min period and then stored in 500 ml plastic and aluminum membrane gas sampling bags. All of the chambers started gas sample collecting at 09:00 (GMT+8), then ended at at 09:30 (GMT+8), to avoid the diurnal fluctuation of N_2_O flux. In order to clarify N_2_O flux without impact of aboveground vegetation at the three sites, N_2_O flux was measured again after aboveground vegetation removed. The N_2_O concentration was then analyzed within one week by gas chromatography (7890A, Agilent, California, USA), equipped with an electron capture detector (ECD) for N_2_O.

### Soil properties

Soil samples were collected in August, 2012. Close to each chamber at the site LIT and CON, soil samples at 0–10, 10–20, 20–30 cm depth were collected. At the site BUR, the chambers were removed after gas sampling. Soil samples from three layers were collected at the place where the chambers were installed to ensure the soil samples reflect the condition of the pika burrows. Because of the burrows were at 10–20 cm soil depth, soil samples from the entrance or near it were collected, representing soil at the 10–20 cm depth at the site BUR.

Soil samples were separated into three sets. The first set of soil was air dried, passed through a 0.18 mm sieve first and then analyzed for soil organic matter (SOM), total N, and total C. The second set of soil was frozen immediately after collecting and reserved for NO_3_-N and NH_4_-N analysis, using a Discrete Auto Analyzer (Smartchem 300, AMS, Italy). The third set was collected by carefully cutting the soil at the edge of the sampling ring, made of stainless steel (5 cm diameter, 5 cm height) for measurement of the bulk density and water content [25]. The SOM of dried samples was measured by the potassium-dichromate oxidation procedure after H_2_SO_4_–HClO_4_ digestion [26]. Total N and total C of dried samples were analyzed using an elemental analyzer (vario MACRO cube, Elementar, Germany).

Since the whole soil samples are collected at very close sites, it is assumed that the densities of soil samples at the same depth are probably consistent. Consequently, soil bulk density and soil water content were taken as proxies to reflect soil aerobic condition.

### Statistical analysis

Nonparametric two-independent-samples test (Mann-Whitney Test) was used to analyze differences of soil properties between the sites at different soil depth. N_2_O flux differences between the sites were also tested this way. The impacts of soil depth and sampling location on soil properties were analyzed by the Repeated-measure ANOVA. The influence of water content on bulk density, SOM, total N, total C, NO_3_-N and NH_4_-N content were analyzed by the Spearman Correlation Analysis. The Spearman Correlation Analysis was also used to analyze the correlation between N_2_O flux and soil traits. All statistical analysis was done with software SPSS Statistics (19.0, IBM, USA).

## Results

### Soil properties

**Fig 1:**
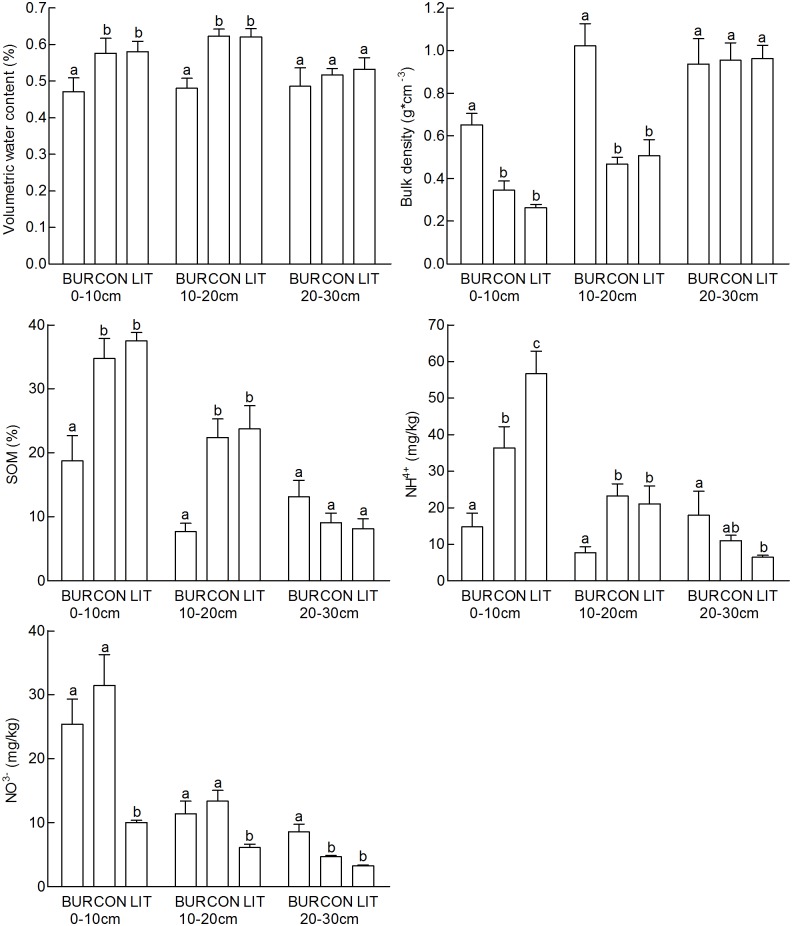
Soil properties at different depth at the sites BUR, CON and LIT. Water content, bulk density, SOM, NH_4_-N content and NO_3_-N content at soil depths of 0–10, 10–20 and 20-30cm in the BUR, CON and LIT sites. With the same lowercase letter did not differ significantly (P>0.05) based on the Mann-Whitney Test.

At a soil depth of 0–10 cm and 10–20 cm, soil water content at the site BUR was significantly lower than that at the site CON, whilst no significantly difference was checked amont soil water content at soil depth of 20-30cm which is located below the cavities of pikas (Fig 1).

Besides the impact of soil depth on soil NH_4_-N, soil depth, sampling location and the interaction between soil depth and sampling location significantly influence the soil properties measured (Table 1).

**Table 1:** Impacts of soil depth and sample location on soil water content, bulk density, SOM, NH_4_-N content and NO_3_-N content.

**Factors**	**Water Content (%)**			Bulk Density (g*cm-3)			**SOM (%)**		
	df	F	P	df	F	P	df	F	P
Location	2	27.759	<0.001*	2	17.583	<0.001*	2	12.663	0.001*
Depth	2	6.711	0.004*	2	6.038	0.007*	2	15.407	<0.001*
Depth× Location	4	6.688	0.001*	4	5.058	0.003*	4	7.406	<0.001*
Factors	NH_4_-N(mg/kg)			NO_3_-N(mg/kg)					
	df	F	P	df	F	P			
Location	2	7.242	0.007*	2	13.801	<0.001*			
Depth	2	2.749	0.081	2	15.428	<0.001*			
Depth× Location	4	10.702	<0.001*	4	5.745	0.004*			

F and *P* values based on repeated-measure ANOVA are given.

The asterisk (*) means significant difference at P < 0.05

Bulk density and SOM showed no significant difference at 20-30cm soil depth in the three sites (Fig 1). At 0-10cm and 10-20cm soil depth, SOM at the site BUR was significantly lower than that at the site CON, and bulk density at the site BUR was significantly higher than that at the site CON. The NH_4_-N content of the soil at the site BUR was significantly lower than that at the site CON at 0-10cm and 10-20cm soil depth, while the soil in the site LIT contained significantly more NH_4_-N than that at the site CON at 0-10cm soil depth. The NO_3_-N content of the soil at the site BUR was significantly higher than that at the site CON at 20-30cm soil depth, while the soil at the site LIT contained significantly less NO_3_-N than that at the site CON at 0-10cm and 10-20cm soil depth.

### N_2_O fluxes

**Fig 2:**
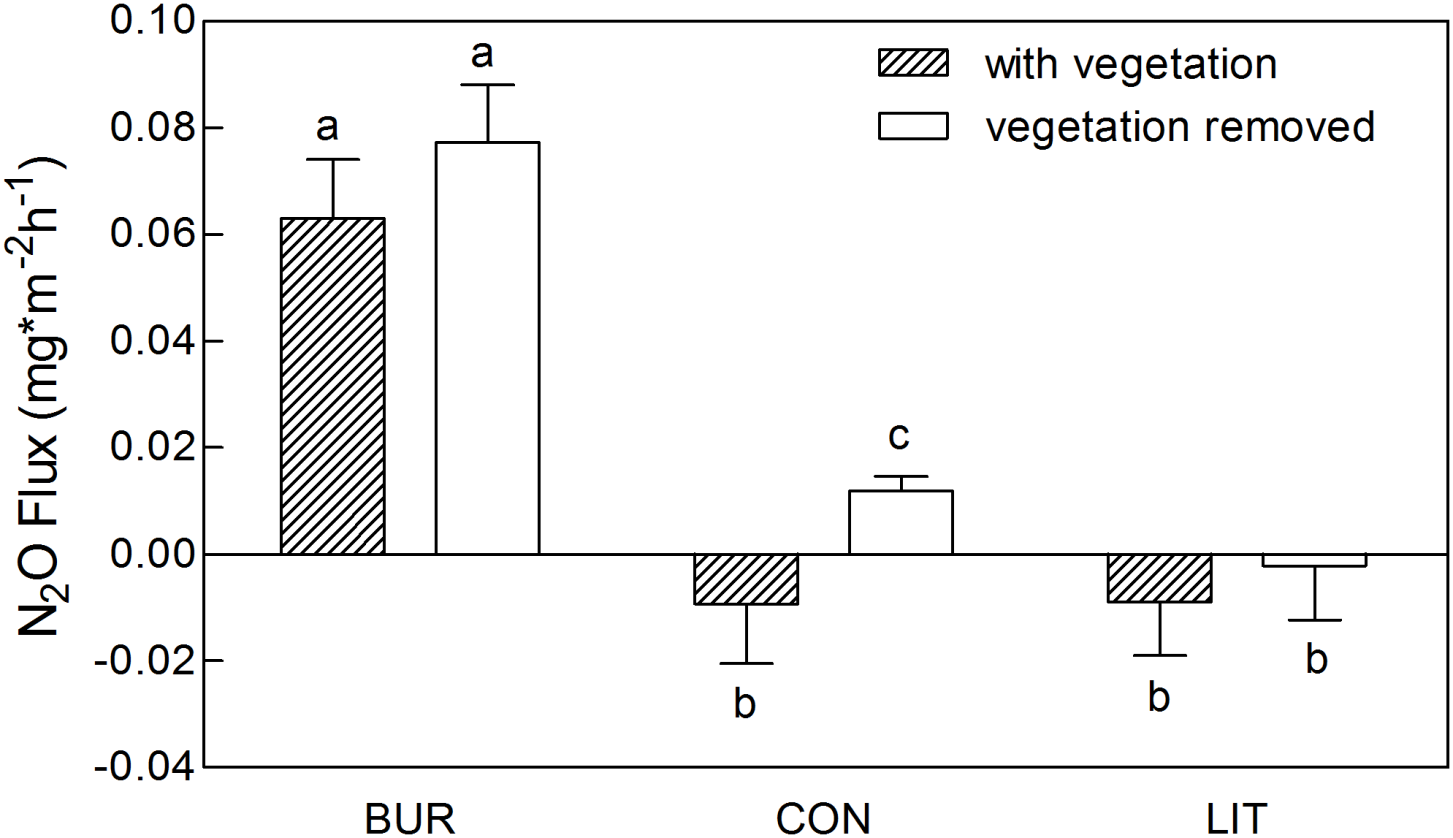
N_2_O fluxes from the sampling sites.

N_2_O flux from the site with burrows (BUR) was significantly higher than that from the control site CON (Fig 2), irrespective of whether the aboveground vegetation was removed or not (Mann-Whitney test, P<0.001 with vegetation, P<0.001 with vegetation removed). The effect of removing vegetation was small compared to the effect of burrows being present in the soil. N_2_O flux from the BUR site increased from 0.063±0.011 mg m^-2^ h^-1^ (mean±SE) to 0.077±0.011 mg m^-2^ h^-1^ after removing the aboveground vegetation. N_2_O flux from the CON and LIT site was -0.009±0.011 mg m^-2^ h^-1^, -0.009±0.010 mg m^-2^ h^-1^, and then increased to 0.012±0.003 mg m^-2^ h^-1^, -0.002±0.010 mg m^-2^ h^-1^ with aboveground vegetation removed, respectively.

N_2_O fluxes were measured at the sites BUR, CON and LIT with aboveground vegetation and aboveground vegetation removed.

The removal of aboveground vegetation at the sampling sites increased N_2_O flux, but only significantly increased the N_2_O flux at the site CON. With aboveground vegetation removed, the site CON changed from being a small N_2_O sink to being a source (Mann-Whitney test, P = 0.005).

### Interactions between soil properties and N_2_O fluxes

Significant correlations were found between N_2_O flux and bulk density, SOM and NH_4_-N content; while N_2_O flux show no significant correlation with volume water content and NO_3_-N (Table 2). N_2_O flux was positively correlated with bulk density, while negatively correlated with SOM and NH_4_-N content.

**Table 2:** Correlation between N_2_O flux and soil properties. The asterisks indicate significant correlation at p<0.05 based on the Spearman’s Correlation Analysis, *r*.

	Water content	Bulk density	SOM	NH_4_-N	NO_3_-N
***r***	-0.38	0.63	-0.57	-0.79	0.23
**Sig**.	0.12	0.005*	0.014*	<0.001*	0.36
**N**	18	18	18	18	17

## Discussion

The Pika’s burrowing transfers the deeper soil to the ground surface during the construction of a network of subterranean burrows. The burrows created by each individual may range from 8 m to 13 m with each branch of the burrow extending 1 m to 5 m [27]. Burrowing activity mixes soil from different soil depth and bring more air into soil, which makes more soil water releasing to the air, then decreases soil water content, especially in the upper layer of soil. The alteration of soil aeration may have also led to changes in soil properties such as the mineralization rate of soil organic matter [28]. In this study, we found that pika’s burrowing significantly reduced soil water content at 0–10 cm and 10–20 cm soil depth (Fig 1). The alteration of soil water content could be also explained by the alteration of other soil properties including bulk density and SOM which shows significant correlations with soil water content (Table 3).

**Table 3:** Relationship between water content and bulk density, SOM, NH4-N, NO3-N content. The asterisks indicate significant correlation at p<0.01(Spearman’s Correlation Analysis *r*).

	Bulk density	SOM	NH_4_-N	NO_3_-N
***r***	-0.573	0.531	0.398	0.107
**Sig**.	<0.001*	<0.001*	0.003*	0.454
**N**	54	54	54	51

Pika activities including daily clearing of the entrance hole and seasonal maintenance of underground structures, such as burrow excavation and wall repair may increase bulk density by squeezing the soil [3], and then causing soil water content altering.

SOM is strongly related to soil water content [29]. Decreasing soil water content can accelerate SOM decomposition. It was reported that decomposition of SOM is greatly reduced in anaerobic environments such as marsh and swampland [30]. In this study, the water content at the site CON and LIT was significantly higher than that of BUR at 0–20 cm soil depth (Fig 1), which presumably made SOM at the site BUR easy to decompose. Pika’s burrowing can also directly promote SOM decomposing by breaking organic matter into smaller particles and making soil fully mixed [31,32].

Nitrification generally occurs in aerobic environments while denitrification occurs in anaerobic environment. As Fig 1 shows, the different soil water content at the sites determined the NH_4_-N content and NO_3_-N content. At the site LIT, higher soil water content resulted in higher NH_4_-N content and lower NO_3_-N content, which is vice versa at the site BUR. NH_3_ volatilization is enhanced in alkaline soil, which accelerate NH_4_-N loss at the site BUR.

The average N_2_O fluxes at the site CON and LIT were -0.009±0.011 mg m^-2^ h^-1^, -0.009±0.010 mg m^-2^ h^-1^, representing weak N_2_O sinks. In previous studies, the average N_2_O fluxes from meadow soil were around 0.03–0.04 mg m^-2^ h^-1^ in Haibei County [33], 0.07±0.11 mg m^-2^ h^-1^ in the Zoige Wetland [34], representing weak N_2_O sources. The differences in N_2_O fluxes between these sites may be induced by the different soil water content. Due to most of precipitation occurring during the growing season under typical Tibetan plateau cold climate, the annual precipitation was taken as proxy to interpret how water content impact N_2_O flux. In 2012, the annual precipitation was 744mm in Zoige, compared with 526mm and 610mm in 2006 and 2007 in Zoige, and 450mm in 2005 in Haibei.

Soil animals are considered as soil ecosystem engineers, as they modify soil structure and interact with microbes through their burrowing activities [35]. N_2_O emissions from soil burrowed by earthworms can be up to three times greater than from control soil [36]. It is concluded that soil disturbance by animals usually alters the structure of the upper soil, and changes the oxidation-reduction condition of the soil. In this study, pika’s burrowing brought deeper soil to the ground surface and exposed soil at surface of burrow to the atmosphere. The soil was thus vulnerable to soil denitrification and carbon oxidation, which was reflected by the decreased SOM and NO_3_-N from the upper soil at the site BUR. N_2_O flux at the site BUR significantly exceeded that at the site CON, suggesting that burrowing significantly increases the N_2_O emission in the Zoige Wetland. Spatial variation of N_2_O fluxes can be explained by the water content and soil nitrate content [37–39]. Due to the lower NO_3_-N content at upper layer of soil, N_2_O flux at the site LIT was much lower than that at the site CON with above-ground vegetation removed from both sites. On the other hand, N_2_O flux from the site BUR was much higher than that from the site CON under same magnitude of soil NO_3_-N content, mainly because of the lower water content at upper layer of soil (Fig 1) resulting more N_2_O produced from nitrification process. However, no significant correlation was found between N_2_O flux and soil water content, NO_3_-N content (Table 2), partly because soil water content and NO_3_-N content together determine N_2_O flux, causing each parameter could not be checked significant correlation with N2O flux.

It is also reported that CO_2_ dissipates into pika tunnels could be easily emitted into the atmosphere through pika holes and then significantly increases the CO_2_ flux from the area of pika burrows [40]. However, when measuring N_2_O flux from pika burrow in this study, it is difficult to distinguish the N_2_O released by underground burrows from the N_2_O flux released by the ground surface, which may amplify the N_2_O flux at the site BUR. The existing data cannot clearly clarify the N_2_O flux caused by pika burrowing, but the magnitude of N_2_O flux from pika burrow can be estimated by taking the density of pika burrow into account. The density of pika burrows around Huahu Lake was about 1100 per hectare (unpublished data). In other region of Qinghai-Tibet Plateau, the burrow density may achieve 1360 per hectare [41]. Consequently, N_2_O flux raised by pika burrowing could not be neglected because of the large magnitude.

The impact of fossorial animals on ecosystem function is beginning to attract more attention [4] and the debate on the role played by fossorial animals in ecosystem continues. To precisely clarify the function of plateau pika on the Qinghai-Tibet Plateau, the process and mechanism of how pika burrowing influences soil properties and greenhouse gas emission should be further investigated.

## Conclusions

In Zoige wetland, the burrowing activity of plateau pika altered the soil structure, resulting in decreasing soil water content, SOM and NH_4_-N content at the upper layer of soil around the burrow. With alteration of soil properties, especially decreasing of soil water content, pika burrow also caused significantly higher N_2_O flux, inducing the meadow from N_2_O sink to N_2_O source. In order to precisely evaluate the impact of pika burrowing on soil traits and N_2_O flux, further work is required to investigate N_2_O flux released by subterranean burrows, mechanism of soil alteration.

## References

[1] Lubbers IM, van Groenigen KJ, Fonte SJ, Six J, Brussaard L, van Groenigen J. Greenhouse-gas emissions from soils increased by earthworms. Nature Climate Change. 2013; 3: 187–194.

[2] Kurek P, Kapusta P, Holeksa J. Burrowing by badgers (Meles meles) and foxes (Vulpes vulpes) changes soil conditions and vegetation in a European temperate forest. Ecological Research. 2014; 29: 1–11.

[3] Hogan BW The plateau pika: A keystone engineer on the Tibetan Plateau. Ph.D. Thesis, Arizona State University. 2010.

[4] Davidson AD, Detling JK, Brown JH. Ecological roles and conservation challenges of social, burrowing, herbivorous mammals in the world’s grasslands. Frontiers in Ecology and the Environment. 2012; 10: 477–486.

[5] Li W, Zhang Y. Impacts of plateau pikas on soil organic matter and moisture content in alpine meadow. Acta theriologica sinica. 2006; 26: 331–337.

[6] Liu W, Xu Q, Wang X, Zhao J, Zhou L. Influence of burrowing activity of plateau pikas (*Ochotona curzoniae*) on nitrogen in soils. Acta Theriologica Sinica. 2010; 30: 35–44.

[7] Yu C, Zhang J, Pang XP, Wang Q, Zhou YP, Guo ZG. Soil disturbance and disturbance intensity: Response of soil nutrient concentrations of alpine meadow to plateau pika bioturbation in the Qinghai-Tibetan Plateau, China. Geoderma. 2017; 307: 98–106.

[8] Liu YS, Fan JW, Harris W, Shao QQ, Zhou YC, Wang N, et al Effects of plateau pika (Ochotona curzoniae) on net ecosystem carbon exchange of grassland in the Three Rivers Headwaters region, Qinghai-Tibet, China. Plant and Soil. 2013; 366: 491–504.

[9] Zhou Y, Li N, Grace J, Yang M, Lu C, Geng XM, et al Impact of Groundwater Table and Plateau Zokors (*Myospalax baileyi*) on Ecosystem Respiration in the Zoige Peatlands of China. PLoS ONE. 2014; 9: e115542 10.1371/journal.pone.0115542 25542023 PMC4277300

[10] Zhou M, Zhu B, Wang S, Zhu X, Vereecken H, Brüggemann N. Stimulation of N2O emission by manure application to agricultural soils may largely offset carbon benefits: a global meta-analysis. Global Change Biology. 2017; 23(10): 4068–4083. 10.1111/gcb.13648 28142211

[11] Myhre G, Shindell D, Bréon F-M, Collins W, Fuglestvedt J, Huang J. Anthropogenic and Natural Radiative Forcing. In: Stocker TF, Qin D, Plattner G-K, Tignor M, Allen SK et al., editors. Climate Change 2013: The Physical Science Basis Contribution of Working Group I to the Fifth Assessment Report of the Intergovernmental Panel on Climate Change. Cambridge, United Kingdom and New York, NY, USA: Cambridge University Press; 2013. pp. 714.

[12] Hartmann DL, Tank AMGK, Rusticucci M, Alexander LV, Brönnimann S, Charabi Y. Observations: Atmosphere and Surface. In: Stocker TF, Qin D, Plattner G-K, Tignor M, Allen SK et al., editors. Climate Change 2013: The Physical Science Basis Contribution of Working Group I to the Fifth Assessment Report of the Intergovernmental Panel on Climate Change. Cambridge, United Kingdom and New York, NY, USA: Cambridge University Press; 2013. pp. 167–168.

[13] Burgos M, Sierra A, Ortega T, Forja JM. Anthropogenic effects on greenhouse gas (CH_4_ and N_2_O) emissions in the Guadalete River Estuary (SW Spain). The Science of the total environment. 2015; 503–504: 179–189. 10.1016/j.scitotenv.2014.06.038 24993513

[14] Brown JR, Blankinship JC, Niboyet A, van Groenigen KJ, Dijkstra P, Le Roux X. Effects of multiple global change treatments on soil N2O fluxes. Biogeochemistry. 2012; 109: 85–100.

[15] Heincke M, Kaupenjohann M. Effects of soil solution on the dynamics of N2O emissions: a review. Nutrient Cycling in Agroecosystems. 1999; 55: 133–157.

[16] Zhang W, Liu C, Zheng X, Fu Y, Hu X, Cao GM. The increasing distribution area of zokor mounds weaken greenhouse gas uptakes by alpine meadows in the Qinghai-Tibetan Plateau. Soil Biology & Biochemistry. 2014; 71: 105–112.

[17] Lipiec J, Brzezinska M, Turski M, Szarlip P, Frac M. Wettability and biogeochemical properties of the drilosphere and casts of endogeic earthworms in pear orchard. Soil & Tillage Research. 2015; 145: 55–61.

[18] Smith AT, Badingqiuying, Wilson MC, Hogan BW. Functional-trait ecology of the plateau pika Ochotona curzoniae (Hodgson, 1858) in the Qinghai-Tibetan Plateau ecosystem. Integrative Zoology. 2018. 10.1111/1749-4877.12300 29316275

[19] Zhang R. Zoogeography of China. Beijing: Science Press; 1999.

[20] Qu J, Li W, Yang M, Ji W, Zhang Y. Life history of the plateau pika (Ochotona curzoniae) in alpine meadows of the Tibetan Plateau. Mammalian Biology—Zeitschrift für Säugetierkunde. 2013; 78: 68–72.

[21] Zhou H, Zhao X, Tang Y, Gu S, Zhou L. Alpine grassland degradation and its control in the source region of the Yangtze and Yellow Rivers, China. Grassland Science. 2005; 51: 191–203.

[22] Smith AT, Foggin JM. The plateau pika (*Ochotona curzoniae*) is a keystone species for biodiversity on the Tibetan plateau. Animal Conservation. 1999; 2: 235–240.

[23] Chen H, Wu N, Yao SP, Gao YH, Zhu D, Wang YF, et al High methane emissions from a littoral zone on the Qinghai-Tibetan Plateau. Atmospheric Environment. 2009; 43: 4995–5000.

[24] Yang M, Geng XM, Grace J, Jia YF, Liu YZ, Jiao SW, et al N_2_O fluxes from the littoral zone of a Chinese reservoir. Biogeosciences. 2015; 12: 4711–4723.

[25] Lu X. Observation Methods in Wetland Ecosystem. Beijing: China Environmental Science Press; 2005.

[26] Semenov M, Kravchenko I, Semenov V, Kuznetsova T, Dulov L, Udal’tsov SN, et al Carbon dioxide, methane, and nitrous oxide fluxes in soil catena across the right bank of the Oka River (Moscow oblast). Eurasian Soil Science. 2010; 43: 541–549.

[27] Wei XH, Li S, Yang P, Cheng HS. Soil erosion and vegetation succession in alpine Kobresia steppe meadow caused by plateau pika—A case study of Nagqu County, Tibet. Chinese Geographical Science. 2007; 17: 75–81.

[28] Tan X, Chang SX. Soil compaction and forest litter amendment affect carbon and net nitrogen mineralization in a boreal forest soil. Soil and Tillage Research. 2007; 93: 77–86.

[29] Hudson BD. Soil organic matter and available water capacity. Journal of Soil and Water Conservation. 1994; 49: 189–194.

[30] Luo Y, Zhou X. Soil respiration and the environment: Academic Press; 2006.

[31] Martin BG. The role of small ground-foraging mammals in topsoil health and biodiversity: Implications to management and restoration. Ecological Management & Restoration. 2003; 4: 114–119.

[32] Yoshihara Y, Okuro T, Buuveibaatar B, Undarmaa J, Takeuchi K. Clustered animal burrows yield higher spatial heterogeneity. Plant ecology. 2010; 206: 211–224.

[33] Du Y, Cui Y, Xu X, Liang D, Long R, Cao GM. Nitrous oxide emissions from two alpine meadows in the Qinghai–Tibetan Plateau. Plant and Soil. 2008; 311: 245–254.

[34] Chen H, Wang M, Wu N, Wang Y, Zhu D, Gao YH, et al Nitrous oxide fluxes from the littoral zone of a lake on the Qinghai-Tibetan Plateau. Environmental monitoring and assessment. 2011; 182: 545–553. 10.1007/s10661-011-1896-y 21327481

[35] Lavelle P. Soil function in a changing world: the role of invertebrate ecosystem engineers. European Journal of Soil Science. 1997; 33: 159–193.

[36] Elliott PW, Knight D, Anderson JM. Variables controlling denitrification from earthworm casts and soil in permanent pastures. Biology & Fertility of Soils. 1991; 11: 24–29.

[37] Audet J, Elsgaard L, Kjaergaard C, Larsen SE, Hoffmann CC. Greenhouse gas emissions from a Danish riparian wetland before and after restoration. Ecological Engineering. 2013; 57: 170–182.

[38] Huang XY, Zhang TP, Li WJ, Wan JQ, Dahlgren RA. Spatial variations in the N2O emissions and denitrification potential of riparian buffer strips in a contaminated urban river. Chemistry and Ecology. 2013; 29: 529–539.

[39] Lan T, Han Y, Roelcke M, Nieder R, Cai Z. Processes leading to N2O and NO emissions from two different Chinese soils under different soil moisture contents. Plant and Soil. 2013; 371: 611–627.

[40] Yu Q, Chen J, Yi S. Plateau pikas burrowing activity accelerates ecosystem carbon emission from alpine grassland on the Qinghai-Tibetan Plateau. Ecological Engineering. 2015; 84: 287–291.

[41] Sun FD, Long Rj, Lu CX. Effects of plateau pikas (Ochotona curzoniae) burrow densities on plant community composition and population diversity in alpinemeadow. Journal of Arid Land Resources and Environment. 2010; 24(7): 181–186.

